# High-Temperature Molecular Beam Epitaxy of Hexagonal Boron Nitride with High Active Nitrogen Fluxes

**DOI:** 10.3390/ma11071119

**Published:** 2018-06-30

**Authors:** Tin S. Cheng, Alex Summerfield, Christopher J. Mellor, Andrei N. Khlobystov, Laurence Eaves, C. Thomas Foxon, Peter H. Beton, Sergei V. Novikov

**Affiliations:** 1 School of Physics and Astronomy, University of Nottingham, Nottingham NG7 2RD, UK; tin.cheng@nottingham.ac.uk (T.S.C.); alex.summerfield@nottingham.ac.uk (A.S.); chris.mellor@nottingham.ac.uk (C.J.M.); laurence.eaves@nottingham.ac.uk (L.E.); tom.foxon@nottingham.ac.uk (C.T.F.); peter.beton@nottingham.ac.uk (P.H.B.); 2 School of Chemistry, University of Nottingham, Nottingham NG7 2RD, UK; andrei.khlobystov@nottingham.ac.uk

**Keywords:** UKNC, III-nitrides, nanostructures, MBE, hexagonal boron nitride

## Abstract

Hexagonal boron nitride (hBN) has attracted a great deal of attention as a key component in van der Waals (vdW) heterostructures, and as a wide band gap material for deep-ultraviolet devices. We have recently demonstrated plasma-assisted molecular beam epitaxy (PA-MBE) of hBN layers on substrates of highly oriented pyrolytic graphite at high substrate temperatures of ~1400 °C. The current paper will present data on the high-temperature PA-MBE growth of hBN layers using a high-efficiency radio-frequency (RF) nitrogen plasma source. Despite more than a three-fold increase in nitrogen flux with this new source, we saw no significant increase in the growth rates of the hBN layers, indicating that the growth rate of hBN layers is controlled by the boron arrival rate. The hBN thickness increases to 90 nm with decrease in the growth temperature to 1080 °C. However, the decrease in the MBE temperature led to a deterioration in the optical properties of the hBN. The optical absorption data indicates that an increase in the active nitrogen flux during the PA-MBE process improves the optical properties of hBN and suppresses defect related optical absorption in the energy range 5.0–5.5 eV.

## 1. Introduction

Hexagonal boron nitride (hBN) is a van der Waals (vdW) material that has been exploited either as an insulating layer, or as a quantum tunnel barrier in a diverse range of two-dimensional (2D) heterostructures and functional devices [1,2,3]. It is also attracting much attention as a wide band gap semiconductor material for deep-ultraviolet (DUV) applications [4,5,6]. There are now attempts world-wide to develop a reproducible technology for the epitaxial growth of large area high-quality hBN layers by chemical vapour deposition (CVD) [7,8,9], metal-organic chemical vapor deposition (MOCVD) [4,5,10,11], and molecular beam epitaxy (MBE) [6,12,13,14,15,16,17,18,19,20,21,22,23,24,25,26,27,28,29].

We have recently demonstrated high-temperature (HT) plasma-assisted MBE (PA-MBE) of hBN layers on both sapphire and highly oriented pyrolytic graphite (HOPG) substrates [22,26,28]. Our results demonstrated that by growing hBN using PA-MBE at HOPG substrate temperatures of ~1400 °C it is possible to produce monolayer and/or few-layer thick boron nitride with atomically flat hBN surfaces, which are essential for future applications. The hBN coverage can be reproducibly controlled by the growth time, substrate temperature and boron to nitrogen flux ratios. We have achieved thicker hBN layers at higher B:N flux ratios. We have demonstrated an increase of the hBN layer thickness by decreasing the growth temperature [28]. However, decreasing the epitaxy temperature below 1250 °C, rapidly degraded the optical properties of the hBN layers.

We have studied the optical properties of MBE-grown hBN using spectroscopic ellipsometry and photoluminescence (PL) [26]. The PL spectrum of hBN, grown on both sapphire and HOPG, is dominated by a strong emission in the DUV, centred around 5.4 eV with a secondary shoulder at ~5.5 eV [26]. These DUV peaks were attributed to the presence of point defects [26,30] and they were also observed in layers grown by MOCVD [5,31] and in bulk hBN crystals [30,32]. In hBN layers grown by MOCVD, increasing the ammonia flow led to the suppression of the DUV lines at 5.3 and 5.5 eV, suggesting the involvement of nitrogen vacancies in the formation of this defect [5,31]. Therefore, it is now timely to explore a similar approach in MBE by increasing the active nitrogen overpressure during PA-MBE growth of hBN layers in order to suppress the formation of nitrogen vacancies in hBN layers.

Our previous experiments on PA-MBE of hBN layers used a standard nitrogen Veeco radio-frequency (RF) plasma source, and nitrogen flow rates of up to two standard cubic centimetres per minute (sccm), to produce a flux of active nitrogen [22,26,28]. Recently, there have been active efforts from the main MBE companies to increase the efficiency of their nitrogen RF plasma sources and to achieve higher MBE growth rates for AlGaInN-based alloys [33,34,35,36,37]. The latest model of the Riber RF plasma source produced growth rates for GaN layers up to 7.6 µm/h using nitrogen flow rates of 25 sccm [34]. The new Veeco’s high-efficiency RF source produced GaN growth rates of up to 9.8 µm/h using a nitrogen flow of 20 sccm [36].

In this paper we present our recent results on the high-temperature PA-MBE growth of hBN layers using a high-efficiency RF plasma source with high active nitrogen fluxes.

## 2. Materials and Methods

The growth of hBN layers was studied using a custom-designed Veeco GENxplor™ MBE system (St. Paul, MN, USA) modified to achieve growth temperatures of up to 1850 °C under ultra-high vacuum conditions on rotating substrates with diameters of up to 3 inches. Details of the MBE system can be found elsewhere [22,28]. Consistent with our previous studies, we used thermocouple readings to measure the substrate temperature [22,28]. We have used a Veeco high-temperature effusion cell for evaporation of boron and a high-efficiency Veeco Gazelle RF plasma source (St. Paul, MN, USA) for active nitrogen, similar to one described above [36]. Boron has two stable isotopes: ^11^B (80.1%) and ^10^B (19.9%) [38]. In our MBE of hBN layers, we have used high-purity (5 N) elemental boron, which contains this natural mixture of these ^11^B and ^10^B isotopes. All hBN layers investigated in this paper were grown using a fixed RF power of 550 W and a nitrogen (N_2_) flow rate of 7 sccm. We used 10 × 10 mm^2^ HOPG substrates with a mosaic spread of 0.4°. The HOPG substrates were prepared by exfoliation using adhesive tape to obtain a fresh graphite surface for epitaxy. After exfoliation, the HOPG substrates were additionally cleaned in toluene overnight and annealed at 200 °C in a H_2_:Ar (5%:95%) gas flow for 4 h to remove any tape residue from the exfoliation process, as described previously [22,28].

Topographic images of the hBN layers were acquired after growth with amplitude-modulated tapping mode atomic force microscopy (AC-AFM, in repulsive mode, in ambient conditions with an Asylum Research Cypher-S AFM (Santa Barbara, CA, USA) using Multi75A1-G (Budget Sensors, stiffness ~3 N/m) cantilevers. AFM image processing and analysis was performed using Gwyddion [39].

Variable angle spectroscopic ellipsometry was performed using a M2000-DI instrument produced by J.A. Woollam Inc. (Lincoln, NE, USA). The results were collected over a wide wavelength range from 1690 to 192 nm using focusing probes, with an elliptical spot with a minor axis of 200 μm; the major axis of the ellipse depends on the angles of incidence, which were 65°, 60° and 55°. Analysis was carried out using CompleteEASE® software version 5.19 (Lincoln, NE, USA). The existence of mosaic spread in the HOPG substrates requires us to allow a small angular offset (<1°) in the ellipsometric models. A Gaussian oscillator and ultra-violet pole were used to model the optical response of the boron nitride layer. A Tauc-Lorentz oscillator was also tried, but it did not significantly improve the fit and so the results presented here only reflect the Gaussian oscillator, as the model had fewer parameters. For measuring thin hBN layers (<10 nm), correlations occur between the fitting parameters, making it hard to estimate the layer thickness. To ensure that the thicknesses were physically reasonable, the hBN refractive index at 633 nm was constrained to be close to 2.0.

## 3. Results and Discussions

For applications of hexagonal boron nitride in 2D vdW heterostructures, it is important to develop the reproducible MBE technology of monolayer hBN and few-layer hBN with monolayer control of the thickness and atomically flat surfaces. Therefore, it is interesting to examine the influence of higher nitrogen overpressures produced by high-efficiency RF sources on the properties of the hBN monolayers. Previously, we established the MBE growth conditions required to grow hBN layers with a thickness of about one monolayer [22,26,28]. Here we have used similar MBE growth conditions for epitaxy of hBN layer with two different nitrogen RF plasma sources.

**Figure 1:**
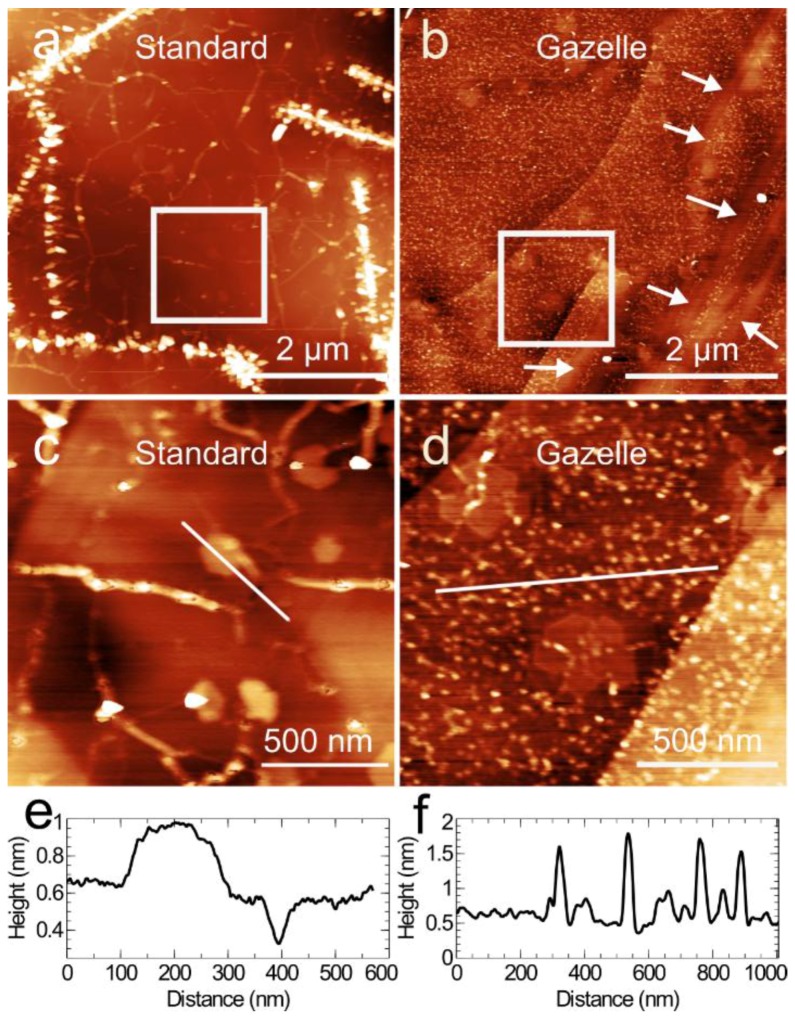
Atomic force microscope (AFM) images of hexagonal boron nitride (hBN) grown on highly oriented pyrolytic graphite (HOPG) at T_S_ = 1390 °C and T_B_ = 1875 °C for 3 h using two different radio-frequency (RF) nitrogen plasma sources: a standard RF source (left column) and a high-efficiency Gazelle RF source (right column). (**a**) Large area AFM image showing a hBN surface grown using standard RF source; bright features correspond to bulky hBN deposits. (**b**) Large area AFM image of hBN grown using the Gazelle RF source. The white arrows indicate step-flow growth from the HOPG terrace steps. (**c**,**d**) Zooms of the areas indicated by the white boxes in (**a**,**b**) respectively. (**e**,**f**) Height profiles along the regions indicated by the white line in (**c**,**d**), respectively.

Figure 1 presents Atomic force microscope (AFM) images of the surface of two hBN layers grown at a substrate temperature (T_S_) of ~1390 °C with a standard Veeco RF nitrogen plasma source (Figure 1a,c) and with the high-efficiency Gazelle source (Figure 1b,d). In both cases, a consistent boron flux was used by maintaining a constant temperature of the boron effusion cell (T_B_) of 1875 °C. Even though the boron fluxes used for the growth of both layers are the same, the morphology of these layers is very different. For the hBN layer grown with the standard plasma source as shown in Figure 1a, we see predominantly flat monolayers of hBN with the boundaries decorated with large 3D features, which nucleate along the HOPG terrace steps, consistent with our previous observations of hBN growth on HOPG under similar conditions [22,26,28]. We observe almost complete coverage of the HOPG substrate with the hBN layer. Some small multilayer (typically bi/tri-layer) regions are also detected, along with small voids in the hBN monolayer, as shown by the high-resolution image in Figure 1b and the associated line profile along the hBN surface (Figure 1e).

For the hBN layer grown with a Gazelle nitrogen source (Figure 1b) there are significant differences in the morphology of the hBN; the growth of bulky 3D deposits of hBN along the HOPG terrace steps is completely suppressed and instead step-flow growth from these regions is achieved. This is indicated by the arrows in Figure 1b. Away from the terrace steps isolated faceted islands of hBN growing on the HOPG terraces are present, indicating that the hBN is not a continuous monolayer. Unlike our previous observations of sub-monolayer hBN coverage in which the regions in between the hBN growth are clean HOPG terraces, we found small deposits with a topographic height of ~1–2 nm, as shown in Figure 1d and the associated height profile in Figure 1f.

**Figure 2:**
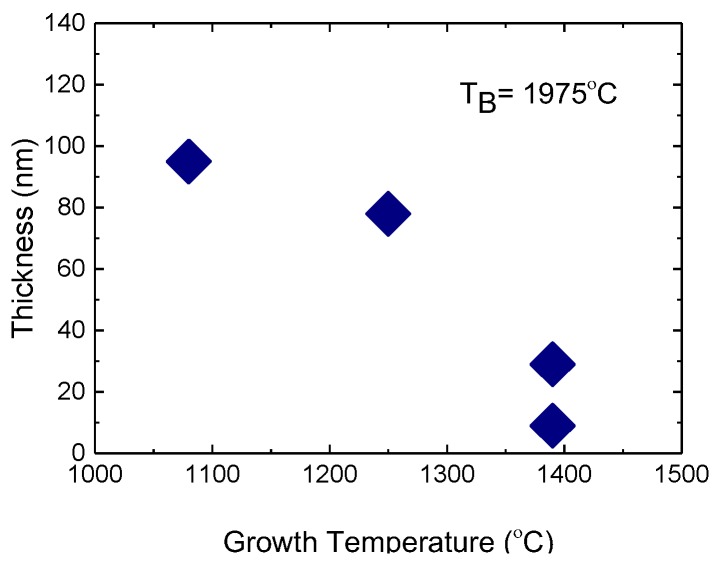
Thickness of hBN layers grown with a Gazelle nitrogen source at different growth temperatures.

In order to establish the influence of the higher active nitrogen fluxes on the growth rate of hBN, we have grown a set of the layers at relatively high boron fluxes where we can measure reproducibly the thicknesses of hBN on the scale of a few tens of nanometres [28]. We have used a high boron source temperature of 1975 °C, growth time of 3 h and growth temperatures between 1080 °C to 1390 °C. The thickness of the hBN layers were measured by variable angle spectroscopic ellipsometry. Figure 2 shows a decrease of the hBN thickness from 90 to 20 nm by increasing the MBE temperature from 1080 °C to 1390 °C. The decrease in the thickness is attributed to an increase in the BN decomposition rate at the growth surface with increase of the growth temperatures, and therefore a gradual increase in the boron re-evaporation rate [28]. The deposition rate of group III-nitride layers in PA-MBE is determined by the balance between the epitaxial growth rate and the decomposition rate [40]. The decomposition rate is negligible at low epitaxial temperatures but must be taken into account when the growth temperature approaches the sublimation temperature of the material. For example, it was demonstrated experimentally that the GaN decomposition rate increases exponentially with the increase in the PA-MBE growth temperatures in the range 750–800 °C [40]. Unfortunately, no data are presently available for the decomposition rate of hBN during PA-MBE, but the trend is probably similar. The hBN layer thicknesses and the increase in thickness with decrease in temperature are similar to the set of hBN layers grown in the same temperature range 1080 °C to 1390 °C with a standard Veeco nitrogen RF plasma source [28]. In the present experiment, we increased the nitrogen flow from the previous 2 sccm [28] to 7 sccm. Despite the more than three-fold increase in nitrogen flux, there was no change in the growth rate of hBN layers. This suggests that the growth rate of hBN layers is controlled by the boron arrival rate and that all our layers grow under strongly N-rich conditions. This is in stark contrast to the standard group-III-rich optimum PA-MBE conditions required for the growth of high-quality AlGaInN layers [41]. However, layer-by-layer growth has also been observed for PA-MBE growth of GaN under strongly N-rich conditions at the relatively high epitaxial temperatures of ~780 °C in the GaN thermal decomposition regime [42]. This was explained by the significant increase in the thermally activated surface diffusion even in the absence of any surfactant Ga monolayers on the surface [42]. In our PA-MBE of hBN, the growth temperatures are significantly higher, reaching 1400 °C. There may be some similarities between high-temperature PA-MBE of AlGaInN and even higher temperature PA-MBE of hBN, but more experimental data are needed to make firm conclusions.

**Figure 3:**
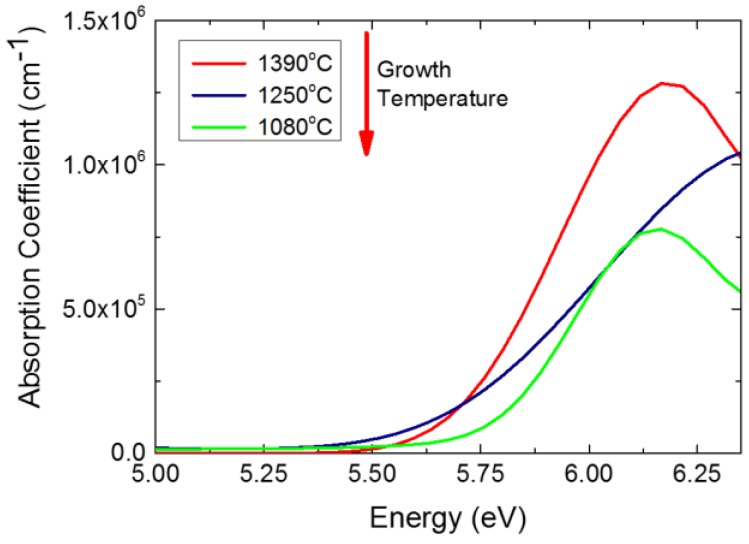
Room temperature optical absorption coefficients for hBN layers grown with Gazelle RF source at three different growth temperatures. The growth time for all layers was 3 h and T_B_ = 1975 °C.

Figure 3 demonstrates absorption coefficient spectra calculated from spectroscopic ellipsometric measurements for the hBN layers grown at the three different MBE temperatures presented in Figure 2. The sharp increase in absorption for the layer grown at 1390 °C suggests a band-gap at around 5.7 eV. With the decrease of the MBE temperature from 1390 °C to 1250 °C, a low energy defect tail below 5.6 eV starts to develop. This suggests an increase in the concentration of the point defects in the hBN layer at the lower growth temperatures. With a decrease of the growth temperature to 1080 °C, the absorption further broadens, suggesting more degradation of the optical properties of hBN. The effect of degradation of absorption with decrease of the MBE temperature is similar to that discussed previously for the hBN layers grown with the standard RF source [28]. This suggests that high-temperature PA-MBE, with substrate temperatures greater than 1250 °C, is a promising approach for the growth of hBN layers with high optical quality. Note that all three hBN samples presented in Figure 3 show lower optical absorption in the range below 5.6 eV, compared to data from hBN samples grown with the standard RF nitrogen plasma source at the same temperatures and reported earlier [28].

**Figure 4:**
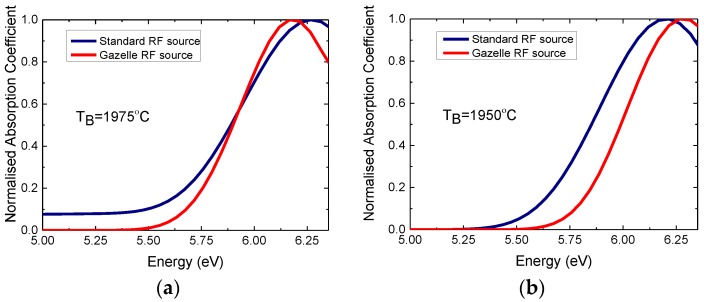
Normalized room temperature optical absorption coefficients for two pairs of hBN layers grown with different boron fluxes T_B_ = 1975 °C (**a**) and TB = 1950 °C (**b**) at a substrate temperature of 1390 °C with two different RF nitrogen sources. The data are normalized by dividing the spectral response by the maximum value in each spectrum.

Figure 4 compares the normalized absorption coefficient spectra for the two pairs of hBN layers, grown with two different nitrogen plasma sources, at the same growth temperatures of 1390 °C with the same boron source temperatures of 1975 °C (Figure 4a) and 1950 °C (Figure 4b). The absorption coefficients have been normalized by dividing the spectral response by the maximum value in each spectrum to make it easier to compare the spectral broadening of the absorption. Figure 4 demonstrates lower absorption in the range 5.0 to 5.7 eV for hBN layers, grown with the high-efficiency Gazelle RF plasma nitrogen source compared to data from the hBN samples grown with the standard RF plasma source. This dependence is seen for both pairs of hBN samples grown with different boron fluxes in Figure 4. The optical absorption data indicates that an increase in the active nitrogen flux during the MBE process results in an improvement of the optical properties of hBN and a suppression absorption in the energy range 5.0–5.5 eV, attributed to point defects related to nitrogen vacancies [5,26,30,31,32]. Further PL studies are required, to support our conclusions and to determine the absorption mechanisms below the hBN band gap.

## 4. Conclusions

We have presented data on the high-temperature PA-MBE of hBN layers using a high-efficiency RF plasma source with high active nitrogen fluxes and a nitrogen flow rate of 7 sccm. Despite the more than three-fold increase in nitrogen flux, we did not see any dramatic increase in the growth rates of hBN layers in comparison with the layers grown with the standard nitrogen RF plasma source. This means that the growth rate of hBN layers is controlled by the boron arrival rate and that all our layers are grown under strongly N-rich conditions. This is in stark contrast to the standard group-III-rich optimum PA-MBE conditions required for the growth of high-quality AlGaInN layers. The morphology of the hBN grown with the high-efficiency RF source is significantly different. We achieved an increase of the hBN thickness by decreasing the MBE temperature. However, decreasing the growth temperature resulted in a deterioration of the optical properties of hBN layers. We have demonstrated lower defect-related absorption in the range 5.0 to 5.5 eV for hBN layers grown with a high-efficiency RF plasma nitrogen source in comparison to data from the hBN samples grown with the standard RF plasma source.

## References

[1] Kubota Y., Watanabe K., Tsuda O., Taniguchi T. (2007). Deep ultraviolet light–emitting hexagonal boron nitride synthesized at atmospheric pressure. Science.

[2] Britnell L., Gorbachev R.V., Jalil R., Belle B.D., Schedin F., Mishchenko A., Georgiou T., Katsnelson M.I., Eaves L., Morozov S.V. (2012). Field-effect tunneling transistor based on vertical graphene heterostructures. Science.

[3] Yang W., Chen G., Shi Z., Liu C.C., Zhang L., Xie G., Cheng M., Wang D., Yang R., Shi D. (2013). Epitaxial growth of single-domain graphene on hexagonal boron nitride. Nat. Mater..

[4] Jiang H.X., Lin J.Y. (2014). Hexagonal boron nitride for deep ultraviolet photonic devices. Semicond. Sci. Technol..

[5] Jiang H.X., Lin J.Y. (2017). Hexagonal boron nitride epilayers: Growth, optical properties and device applications. ECS J. Solid State Sci. Technol..

[6] Laleyan D.A., Zhao S., Woo S.Y., Tran H.N., Le H.B., Szkopek T., Guo H., Botton G.A., Mi Z. (2017). AlN/h-BN heterostructures for Mg dopant-free deep ultraviolet photonics. Nano Lett..

[7] Song L., Ci L., Lu H., Sorokin P.B., Jin C., Ni J., Kvashnin A.G., Kvashnin D.G., Lou J., Yakobson B.I. (2010). Large scale growth and characterization of atomic hexagonal boron nitride layers. Nano Lett..

[8] Kim K.K., Hsu A., Jia X., Kim S.M., Shi Y., Hofmann M., Nezich D., Rodriguez-Nieva J.F., Dresselhaus M., Palacios T. (2012). Synthesis of monolayer hexagonal boron nitride on Cu foil using chemical vapor deposition. Nano Lett..

[9] Caneva S., Weatherup R.S., Bayer B.C., Brennan B., Spencer S.J., Mingard K., Cabrero-Vilatela A., Baehtz C., Pollard A.J., Hofmann S. (2015). Nucleation control for large, single crystalline domains of monolayer hexagonal boron nitride via Si-doped Fe catalysts. Nano Lett..

[10] Li X., Jordan M.B., Ayari T., Sundaram S., El Gmili Y., Alam S., Alam M., Patriarche G., Voss P.L., Salvestrini J.P. (2017). Flexible metal-semiconductor-metal device prototype on wafer-scale thick boron nitride layers grown by MOVPE. Sci. Rep..

[11] Rigosi A.F., Hill H.M., Glavin N.R., Pookpanratana S.J., Yang Y.F., Boosalis A.G., Hu J.N., Rice A., Allerman A.A., Nguyen N.V. (2018). Measuring the dielectric and optical response of millimeter-scale amorphous and hexagonal boron nitride films grown on epitaxial graphene. 2D Mater..

[12] Paisley M.J., Sitar Z., Van B., Davis R.F. (1990). Growth of boron nitride films by gas molecular-beam epitaxy. J. Vac. Sci. Technol. B.

[13] Gupta V.K., Wamsley C.C., Koch M.W., Wicks G.W. (1999). Molecular beam epitaxy growth of boron-containing nitrides. J. Vac. Sci. Technol. B.

[14] Tsai C.L., Kobayashi Y., Akasaka T., Kasu M. (2009). Molecular beam epitaxial growth of hexagonal boron nitride on Ni(111) substrate. J. Cryst. Growth.

[15] Hirama K., Taniyasu Y., Karimoto S., Krockenberger Y., Yamamoto H. (2014). Single-crystal cubic boron nitride thin films grown by ion-beam-assisted molecular beam epitaxy. Appl. Phys. Lett..

[16] Xu Z., Zheng R., Khanaki A., Zuo Z., Liu J. (2015). Direct growth of graphene on in situ epitaxial hexagonal boron nitride flakes by plasma-assisted molecular beam epitaxy. Appl. Phys. Lett..

[17] Nakhaie S., Wofford J.M., Schumann T., Jahn U., Ramsteiner M., Hanke M., Lopes J.M.J., Riechert H. (2015). Synthesis of atomically thin hexagonal boron nitride films on nickel foils by molecular beam epitaxy. Appl. Phys. Lett..

[18] Zuo Z., Xu Z., Zheng R., Khanaki A., Zheng J.-G., Lui J. (2015). In-situ epitaxial growth of graphene/h-BN van der Waals heterostructures by molecular beam epitaxy. Sci. Rep..

[19] Barton A.T., Yue R., Anwar S., Zhu H., Peng X., McDonnell S., Lu N., Addou R., Colombo L., Kim M.J. (2015). Transition metal dichalcogenide and hexagonal boron nitride heterostructures grown by molecular beam epitaxy. Microelectron. Eng..

[20] Tonkikh A.A., Voloshina E.N., Werner P., Blumtritt H., Senkovskiy B., Güntherodt G., Parkin S.S.P., Dedkov Y.S. (2016). Structural and electronic properties of epitaxial multilayer h-BN on Ni (111) for spintronics applications. Sci. Rep..

[21] Xu Z., Khanaki A., Tian H., Zheng R., Suja M., Zheng J.-G., Liu J. (2016). Direct growth of hexagonal boron nitride/graphene heterostructures on cobalt foil substrates by plasma-assisted molecular beam epitaxy. Appl. Phys. Lett..

[22] Cho Y.J., Summerfield A., Davies A., Cheng T.S., Smith E.F., Mellor C.J., Khlobystov A.N., Foxon C.T., Eaves L., Beton P.H. (2016). Hexagonal boron nitride tunnel barriers grown on graphite by high temperature molecular beam epitaxy. Sci. Rep..

[23] Wofford J.M., Nakhaie S., Krause T., Liu X., Ramsteiner M., Hanke M., Riechert H., Lopes J.M.J. (2017). A hybrid MBE-based growth method for large-area synthesis of stacked hexagonal boron nitride/graphene heterostructures. Sci. Rep..

[24] Xu Z., Tian H., Khanaki A., Zheng R., Suja M., Liu J. (2017). Large-area growth of multilayer hexagonal boron nitride on polished cobalt foils by plasma-assisted molecular beam epitaxy. Sci. Rep..

[25] Hirama K., Taniyasu Y., Karimoto S., Yamamoto H., Kumakura K. (2017). Heteroepitaxial growth of single-domain cubic boron nitride films by ion-beam-assisted MBE. Appl. Phys. Express.

[26] Vuong T.Q.P., Cassabois G., Valvin P., Rousseau E., Summerfield A., Mellor C.J., Cho Y., Cheng T.S., Albar J.D., Eaves L. (2017). Deep ultraviolet emission in hexagonal boron nitride grown by high-temperature molecular beam epitaxy. 2D Mater..

[27] Khanaki A., Xu Z., Tian H., Zheng R., Zuo Z., Zheng J.G., Liu J. (2017). Self-assembled cubic boron nitride nanodots. Sci. Rep..

[28] Cheng T.S., Summerfield A., Mellor C.J., Davies A., Khlobystov A.N., Eaves L., Foxon C.T., Beton P.H., Novikov S.V. (2018). High-temperature molecular beam epitaxy of hexagonal boron nitride layers. J. Vac. Sci. Technol. B.

[29] Heilmann M., Bashouti M., Riechert H., Lopes J.M.J. (2018). Defect mediated van der Waals epitaxy of hexagonal boron nitride on graphene. 2D Mater..

[30] Cassabois G., Valvin P., Gil B. (2016). Intervalley scattering in hexagonal boron nitride. Phys. Rev. B.

[31] Du X.Z., Li J., Lin J.Y., Jiang H.X. (2016). The origins of near band-edge transitions in hexagonal boron nitride epilayers. Appl. Phys. Lett..

[32] Watanabe K., Taniguchi T., Kanda H. (2004). Direct-bandgap properties and evidence for ultraviolet lasing of hexagonal boron nitride single crystal. Nat. Mater..

[33] McSkimming B.M., Wua F., Huault T., Chaix C., Speck J.S. (2014). Plasma assisted molecular beam epitaxy of GaN with growth rates >2.6 µm/h. J. Cryst. Growth.

[34] McSkimming B.M., Chaix C., Speck J.S. (2015). High active nitrogen flux growth of GaN by plasma assisted molecular beam epitaxy. J. Vac. Sci. Technol. A.

[35] Novikov S.V., Kent A.J., Foxon C.T. (2017). Molecular beam epitaxy as a growth technique for achieving free-standing zinc-blende GaN and wurtzite Al_x_Ga_1−x_N. Prog. Cryst. Growth Charact. Mater..

[36] Gunning B.P., Clinton E.A., Merola J.J., Doolittle W.A., Bresnahan R.C. (2015). Control of ion content and nitrogen species using a mixed chemistry plasma for GaN grown at extremely high growth rates >9 μm/h by plasma-assisted molecular beam epitaxy. J. Appl. Phys..

[37] Cordier Y., Damilano B., Aing P., Chaix C., Linez F., Tuomisto F., Vennegues P., Frayssinet E., Lefebvre D., Portail M. (2016). GaN films and GaN/AlGaN quantum wells grown by plasma assisted molecular beam epitaxy using a high density radical source. J. Cryst. Growth.

[38] National Institute of Standards and Technology Atomic Weights and Isotopic Compositions for All Elements. https://physics.nist.gov/cgi-bin/Compositions/stand_alone.pl.

[39] Necas D., Klapetek P. (2012). Gwyddion: An open-source software for SPM data analysis. Cent. Eur. J. Phys..

[40] Fernández-Garrido S., Koblmüller G., Calleja E., Speck J.S. (2008). In situ GaN decomposition analysis by quadrupole mass spectrometry and reflection high-energy electron diffraction. J. Appl. Phys..

[41] Heying B., Averbeck R., Chen L.F., Haus E., Riechert H., Speck J.S. (2000). Control of GaN surface morphologies using plasma-assisted molecular beam epitaxy. J. Appl. Phys..

[42] Koblmüller G., Wu F., Mates T., Speck J.S., Fernández-Garrido S., Calleja E. (2007). High electron mobility GaN grown under N-rich conditions by plasma-assisted molecular beam epitaxy. Appl. Phys. Lett..

